# Serum Levels of Vitamin D and Dental Caries in 7-Year-Old Children in Porto Metropolitan Area

**DOI:** 10.3390/nu13010166

**Published:** 2021-01-07

**Authors:** Cátia Carvalho Silva, Sandra Gavinha, Maria Conceição Manso, Rita Rodrigues, Sandra Martins, João Tiago Guimarães, Ana Cristina Santos, Paulo Melo

**Affiliations:** 1 Faculdade de Medicina Dentária, Universidade do Porto (U. Porto), Rua Dr. Manuel Pereira da Silva, 93, 4200-393 Porto, Portugal; pmelo@fmd.up.pt; 2 Faculdade de Ciências da Saúde, Universidade Fernando Pessoa, Praça 9 de Abril, 349, 4249-004 Porto, Portugal; sgavinha@ufp.edu.pt (S.G.); cmanso@ufp.edu.pt (M.C.M.); cmendes@ufp.edu.pt (R.R.); 3 Environmental and Health Research Unit (FP-ENAS), Universidade Fernando Pessoa Energy, 4249-004 Porto, Portugal; 4 Associated Laboratory for Green Chemistry (LAQV/REQUIMTE), Universidade do Porto, 4050-313 Porto, Portugal; 5 Departamento de Patologia Clínica, Centro Hospitalar Universitário de São João, Alameda Prof. Hernâni Monteiro, 4200-319 Porto, Portugal; smvrmartins@gmail.com (S.M.); jtguimar@med.up.pt (J.T.G.); 6 EPIUnit–Instituto de Saúde Pública da Universidade do Porto, Rua das Taipas, 135, 4050-600 Porto, Portugal; acsantos@med.up.pt; 7 Departamento de Biomedicina, Faculdade de Medicina, Universidade do Porto, Alameda Prof. Hernâni Monteiro, 4200-319 Porto, Portugal; 8 Departamento de Ciências da Saúde Pública e Forenses, e Educação Médica da Faculdade de Medicina da Universidade do Porto, Alameda Prof. Hernâni Monteiro, 4200-319 Porto, Portugal

**Keywords:** vitamin D, 25-hydroxyvitamin D, dental caries, children, preventive dentistry, public health, paediatric dentistry

## Abstract

Vitamin D deficiency has been associated with significant changes in dental structures. In children, it can induce enamel and dentin defects, which have been identified as risk factors for caries. This study aimed to assess the association between low serum 25-hydroxyvitamin D (25(OH) D) levels (<30 ng/mL) and the prevalence of caries in the permanent teeth and mixed dentition of 7-year-old children. A sample of 335 children from the population-based birth cohort Generation XXI (Porto, Portugal) was included. Data on children’s demographic and social conditions, health status, dental health behaviours, dental examination including erupted permanent first molars, and blood samples available for vitamin D analysis were collected. Dental outcomes included the presence of caries, including non-cavitated lesions (d_1–6_mft/D_1–6_MFT > 0), and advanced caries (d_3–6_mft/D_3–6_MF > 0). Serum 25(OH) D was measured using a competitive electrochemiluminescence immunoassay protein-binding assay. Bivariate analysis and multivariate logistic regression were used. Advanced caries in permanent teeth was significantly associated with children’s vitamin D levels <30 ng/mL, gastrointestinal disorders, higher daily intake of cariogenic food, and having had a dental appointment at ≤7 years old. Optimal childhood levels of vitamin D may be considered an additional preventive measure for dental caries in the permanent dentition.

## 1. Introduction

Dental caries remains the most prevalent oral disease among children and adolescents, increasing progressively with age [1]. Physical, biological, environmental, and behavioural factors play an important role in dental caries aetiology [2]. The microbiological and environmental factors that cause this dental disease have been extensively studied [1,2,3,4]. In the last few decades, preventive efforts were mainly directed towards behavioural factors; however, attention should also be paid to the hosts’ susceptibility, namely, teeth mineralisation and dental hard-tissue quality. Enamel developmental defects, including molar incisor hypomineralisation [5] and enamel hypoplasia [6,7], result from events in early life and have been identified as major risk factors for dental caries [5,6,8].

Vitamin D plays an important biological role in the human body by helping maintain the normal growth and mineralisation of bone and other calcified tissues, including teeth [7]. The term vitamin D refers to a group of liposoluble secosteroids with an endocrine function. Its two main forms are vitamin D2 (ergocalciferol) and vitamin D3 (cholecalciferol). Both vitamins D2 and D3 are inactive pro-hormones that bind to the vitamin-D binding protein (VDBP) during circulation to be transported to the liver, where the vitamin D 25-hydroxylase enzyme converts them into 25-hydroxyvitamin D (25(OH) D). Then, at the kidneys, the 25-hydroxyvitamin D 1α-hydroxylase enzyme acts on 25(OH) D to transform it into an active metabolite: 1,25-dihydroxyvitamin D (1,25(OH) 2D). That second hydroxylation is regulated by the parathormone based on the concentrations of calcium and phosphate [9,10].

Studies on the molecular basis of vitamin D and vitamin D receptor concluded that vitamin D influences tooth germen formation, helps regulate enamel and dentin formation and maturation, and controls further stages of tooth crown development [11]. In addition to affecting the availability of calcium and phosphate, vitamin D also induces certain antimicrobial peptides, such as defensins, that protect against many pathogens, including oral bacteria [12]. These mechanisms may also explain an increased caries risk in children with low levels of vitamin D [13].

Vitamin D deficiency is evident throughout the European population, with concerning prevalence rates that require action from both public health and clinical perspectives [14]. Several studies reported that a considerable number of healthy European children and adolescents might be vitamin-D deficient, especially in risk groups (i.e., dark skin, insufficient sun exposure, obesity, chronic liver diseases, chronic intestinal diseases, chronic renal diseases, and the use of certain drugs) [9]. The current recommendations on children’s vitamin D reference values seem to agree on a cut-off serum concentration of 20 ng/mL for sufficient vitamin D levels [15]. However, these guidelines are exclusively based on vitamin D’s skeletal action, mainly on preventing nutritional rachitis. This subject is still very controversial, and the Institute of Medicine and the Endocrine Society have indicated different reference cut-off values for 25-hydroxyvitamin D (25(OH) D) sufficiency: 20 ng/mL and 30 ng/mL, respectively [16,17].

Numerous epidemiologic observational studies have shown that vitamin D status in childhood and adolescence may play an important role in the occurrence of dental caries. However, the evidence from recent observational and interventional studies is conflicting, with some studies showing higher caries in children with lower vitamin D levels [18,19,20,21] and others finding no association [22,23,24]. If enamel developmental defects play a relevant role for the higher caries rates, the critical period will be during pregnancy or early life, when tooth formation is most active [5]. Adequate vitamin D early in life may play a role in preventing dental caries in permanent teeth [25]. During pregnancy, prenatal vitamin D levels may influence the primary dentition and the development of early childhood caries [26]. A recently published study on the effect of vitamin-D supplementation during pregnancy on the umbilical cord 25(OH) D levels, and consequently on dental caries in children, found no direct association between supplementation and a lower risk of severe early childhood caries. Nonetheless, they observed that children with a higher concentration of 25(OH) D had a significantly lower score of decayed teeth [27].

Currently, even though dental caries have been related to vitamin D [18,19,20,28,29], there is no clear consensus on this association either in the primary or permanent dentition. A recently published systematic review reported several factors for this, namely related to the heterogeneity in the dental outcomes’ assessment and cut-off points for caries definition, the diversity in the sample study, the methodology for evaluating vitamin D levels and their cut-offs, and the lack of statistical models adjusted for the main confounders of the dental caries/vitamin D association [30]. Therefore, the relationship between children’s vitamin D levels and dental caries is worth investigating following strict methodology regarding intervening factors to find a possible cause-effect relationship.

The purpose of this study was to comprehensively assess the association between low serum 25(OH) D levels (<30 ng/mL) and the prevalence of dental caries in permanent teeth and mixed dentition in 7-year-old children from the population-based cohort Generation XXI.

## 2. Materials and Methods

### 2.1. Study Design and Participants

The sample was obtained from the population-based cohort Generation XXI, which was assembled in the five level-III public maternity units in the Porto Metropolitan Area (Northern Portugal) during 2005–2006 [31]. Generation XXI participants were recruited according to the following eligibility criteria: mothers living in one of the six municipalities of the Porto metropolitan area, delivering at the public maternities that covered those municipalities and giving birth to live babies with a gestational age >24 weeks. At enrolment, the maternity units were responsible for 91.6% of the deliveries in the whole eligible population.

A total of 8647 children and 8495 mothers were enrolled in Generation XXI at baseline [32]. The follow-ups of the entire cohort occurred between April 2009 and July 2011, April 2012 and April 2014, and July 2015 and July 2017, when children were 4, 7, and 10 years of age, respectively. Trained interviewers conducted face-to-face interviews and applied structured questionnaires at baseline and in the follow-up evaluations to collect data on demographic and social conditions, lifestyle, children’s health status, and objective anthropometric measures [32].

At the second follow-up, all Generation XXI 7-year-old children were invited for a dental examination appointment. In this follow-up, 908 children underwent a dental evaluation, and 4595 had blood samples collected. The present study considered a subsample of 3357-year-old children whose permanent first molars had erupted at the time and who had blood samples available for vitamin D analysis. However, not all mothers’ and children’s characteristics were registered for every subject, so the number of subjects for each variable varies slightly.

The characteristics of the children who attended the dental visit and of their mothers were compared with the remaining cohort evaluated at baseline (Table S1). The comparison showed that the children in the present sample had a higher gestational age than the children in the remaining cohort (95.6% vs. 90.6%, *p* = 0.006). No significant differences were found regarding the mothers’ age and education, the monthly income and the children’s birth weight.

### 2.2. Data Collection

Information on the children’s and mothers’ socioeconomic and demographic characteristics, health history, and lifestyle was collected at birth and at the 7-year follow-up, using structured questionnaires applied to the child’s caregiver. Some of the variables were grouped or recoded.

The following variables from the baseline evaluation were used in the present study: the mother’s age (continuous variable) and education level (≤9 years, 10–12 years or >12 years); and the child’s gestational age (<37 weeks or ≥37 weeks) and birth weight (<2500 g, 2500–3800 g or >3800 g). The following variables from the 7-year follow-up were used in this study: the child’s sex (male or female); the household income (≤1000 €, 1001–1500 € or >1500 €); the child’s conditions or diseases (gastrointestinal, bone, muscle, and joint disorders, kidney diseases, growth and liver disorders, epilepsy, cerebral palsy, congenital malformations, and number of bone fractures), when present; the child’s vitamin and drugs intake; and the child’s activities (minutes a week spent reading, watching TV, and doing outdoor activities). This study also used the anthropometric measures collected. The children’s body mass index (BMI) was classified according to standard age- and sex-specific BMI z-scores developed by the World Health Organisation (WHO) [33].

Trained interviewers applied a food frequency questionnaire to evaluate the children’s diet. The parents/caregivers were asked how many times, on average, their child had consumed each of several food items in the previous 6 months: “≥4 times per day;” “2–3 times per day;” “once a day;” “5–6 times per week;” “2–4 times per week;” “once a week;” “1–3 times per month;” “<1 a month;” or “never.” All consumption frequencies were converted into daily frequencies (e.g., once a week was converted into 1/7 days = 0.14 times per day), as previously described [34]. There were two food groups defined under two new variables called “cariogenic foods” (ice cream, breakfast cereals, crackers, cookies, sweet pastry, chocolate, sugar and candies) and “cariogenic drinks” (chocolate milk, sweetened carbonated drinks and other sweetened drinks). Both these variables were analysed as continuous variables.

### 2.3. Dental Examination

The entire cohort was invited to participate in the 7-year follow-up evaluation, and 81% of the children were re-evaluated. As part of the physical evaluation, the children were invited for an oral examination. The 7-year-olds are an important age group because the dentist can assess dental caries not only in the primary dentition but also in the first permanent teeth, which have a different exposure time to other caries risk factors.

At this visit, trained dentists applied a questionnaire to the children’s parents/caregivers on toothbrushing frequency and other dental health-related behaviours, including if the child had already attended a dental appointment (yes/no). The parents/caregivers were asked how many times per day, on average, did their child brush their teeth: “<1 time per week;” “1–2 times per week;” “3–6 times per week;” “once a day;” “2 times per day: 1 at bedtime;” “2 times per day: none at bedtime;” “≥3 times per day: one at bedtime and the others after meals;” “≥3 times per day: none at bedtime or after meals.” The children’s toothbrushing frequencies were converted into three groups: <1 time per day, once a day, and ≥2 times per day.

Furthermore, four trained, calibrated dentists examined the children in a standard chair with a halogen lamp, using a dental mirror and a probe. Plaque removal was also performed, using a sterile gauze to remove it and dry the tooth surfaces, for examining caries lesions according to the International Caries Detection and Assessment System II (ICDAS II) criteria [35,36]. No additional detection methods, including radiographs, were used. The intra- and inter-examiner calibration consisted of a session including an e-learning programme, a theoretical course with images, and a training observation of patients of the same age against a gold standard. To assess consistency between observations, each examiner repeated one of every ten clinical observations during the recording stage. The dentists’ intra- and inter-examiner calibration showed a linear weighted kappa of 0.80 and 0.75, respectively, for ICDAS II with a good agreement [37].

The caries status was determined using the decayed, missing and filled teeth index for the primary (dmft) and permanent dentitions (DMFT), based on the WHO standard methodology [38], and additionally including the incipient lesions in the decayed component. Advanced caries lesions (ICDAS II codes 3–6) were distinguished from initial non-cavitated caries lesions (ICDAS II codes 1–2). Based on the clinical data obtained, two main outcome variables were created for caries. The first was “dental caries status,” defined as the presence or absence of dental caries, including initial non-cavitated caries, in permanent teeth (ICDAS II 1-6 decayed, missing and filled permanent teeth: D_1–6_MFT), and mixed dentition (d_1–6_mft and D_1–6_MFT). A d_1–6_/D_1–6_ lesion was recorded when the tooth showed a first (1) or distinct (2) visual change in enamel, a localised enamel breakdown due to caries with no visible dentine underlying shadow (3), an underlying dark shadow from dentin with or without localised enamel breakdown (4), a distinct cavity with visible dentine (5), or an extensive distinct cavity with visible dentin (6) [35,36]. The second outcome was “advanced dental caries,” defined as the presence or absence of advanced caries lesions in permanent teeth (ICDAS II 3–6 decayed, missing and filled permanent teeth: D_3–6_MFT) and in mixed dentition (d_3–6_mft and D_3–6_MFT). A d_3–6_/D_3–6_ lesion was recorded using codes (3), (4), (5) and (6), as previously described.

The d_1–6_mft/D_1–6_MFT scores were determined by counting the number of carious teeth in the mixed and permanent teeth and applying the following categories, according to the ICDAS II codes: sound surfaces (ICDAS II 00, 10, 20), decayed surfaces (ICDAS II 01–06, 11–16, 21–26, 31–36, 41–46, 51–56, 61–66, 71–76, 81–86), filled surfaces (ICDAS II 30, 40, 50, 60, 70, 80), and missing tooth surfaces due to caries (ICDAS II 97). The d_3–6_mft/D_3–6_MFT scores were determined based on the following categories: sound surfaces (ICDAS II 00, 01, 02, 10, 11, 12, 20, 21, 22), decayed surfaces (ICDAS II 03–06, 13–16, 23–26, 33–36, 43–46, 53–56, 63–66, 73–76, 83–86), filled surfaces (ICDAS II 30, 40, 50, 60, 70, 80), and missing tooth surfaces due to caries (ICDAS II 97). If one tooth had one surface decayed and another filled, it was considered as decayed.

Children with at least one tooth affected by caries in mixed dentition or permanent teeth (d_1–6_mft/D_1–6_MFT > 0 or D_1–6_MFT > 0) were considered to have dental caries. Children with at least one tooth affected by advanced caries lesion in mixed dentition or permanent teeth (d_3–6_mft/D_3–6_MFT > 0 or D_3–6_MFT > 0) were considered to have advanced dental caries.

### 2.4. Serum 25-Hydroxyvitamin D

The 7-year-old children’s vitamin D status was determined by analysing 25(OH) D concentrations in the serum samples collected during the clinical visit. All the samples were analysed at the central laboratory of the São João Hospital in Porto, Portugal. Serum 25(OH) D was determined by a competitive electrochemiluminescence immunoassay protein-binding assay using an Elecsys Cobas^®^ e411 automated analyser (Roche, Roche Diagnostics GmbH, Mannheim, Germany). This assay uses a VDBP as capture protein, which binds to both forms of vitamin D: 25(OH) D2 and 25(OH) D3. Thus, in this paper, the terms ‘vitamin D’ and ‘25(OH) D’ refer to both forms: 25(OH) D2 and 25(OH) D3. The specific terms for vitamin D2 and D3 are used to refer to the corresponding individual form.

The 25(OH) D cut-offs established by the Clinical Guidelines Subcommittee of the Endocrine Society were followed, considering the absence of a global consensus on the 25(OH) D concentration that defines vitamin D deficiency and “adequate” 25(OH) D levels for extra-skeletal functions [39]. Thus, vitamin D adequacy was classified according to the following 25(OH) D cut-off levels: deficiency, ≤20 ng/mL; insufficiency, 21–29 ng/mL; and sufficiency, ≥30 ng/mL [17]. These were later dichotomised into adequate (≥30 ng/mL) and not adequate (<30 ng/mL), for statistical analysis purposes. The season of blood sample collection was also considered, as it may affect the children’s vitamin D concentrations [40]. For that purpose, the year was divided into two seasons: summer (April to September) and winter (October to March).

### 2.5. Statistical Analysis

Descriptive statistics included frequencies (counts and percentages) for qualitative variables and median values and interquartile range (1st and 3rd quartiles) for quantitative data. There were four binary (presence or absence) outcome variables for caries derived from the ICDAS II index: non-cavitated lesions in (1) permanent teeth (D_1–6_MFT) and (2) mixed dentition (d_1–6_mft and D_1–6_MFT), and advanced dental caries in (3) permanent teeth (D_3–6_MFT) and (4) mixed dentition (d_3–6_mft and D_3–6_MFT). We evaluated the dose-response relationship between 25(OH) D levels and children’s activities (weekly minutes spent reading, watching TV, and doing outdoor activities) using Spearman’s correlation coefficient. The bivariate analysis included the chi-square or Fisher’s exact test to determine the association of each qualitative independent variable with the dental caries status and the advanced dental caries. Median birth weight and median cariogenic food and drinks intake of children with dental caries status and advanced dental caries were compared using the Mann–Whitney test for independent samples.

The association between vitamin D and dental caries status and vitamin D and advanced dental caries was examined based on crude odds ratio (OR) and 95% confidence intervals (CIs) from logistic regression. For theoretical reasons [40], the potential effect of the season of blood collection on the children’s levels of vitamin D was also assessed by including an interaction term (vitamin D < 30 ng/mL × winter season) in the models. Further multivariate logistic regression analyses were performed to evaluate the adjusted OR and 95% CIs for dental caries status and advanced dental caries. Variables with a *p* < 0.20 in the bivariate analysis regarding dental caries status and advanced dental caries were included in the first step of the model and backward stepwise Wald elimination was performed (*p* < 0.05 for covariate inclusion and *p* > 0.20 for exclusion). The models were adjusted for maternal education in years (≤9, 10–12 or >12 years) and the season of blood sample collection (summer and winter) was tested as an interaction variable. The variable family household income was excluded in these models due to the evidence of multi-collinearity with maternal education.

A statistical significance of *p* < 0.05 was considered in all analyses. Statistical analyses were conducted using the statistical software package IBM SPSS Statistics v25 (IBM Corp. released 2017, Armonk, NY, USA: IBM Corp.)

### 2.6. Ethical Consideration

The project Generation XXI was conducted according to the Declaration of Helsinki. The Ethical Committee of the São João Hospital/Faculty of Medicine of the University of Porto approved all procedures involving human subjects/patients. The Portuguese Authority of Data Protection also approved this study (n° 5833/2011). The parents or legal tutors of each participant received an explanation on the purpose and design of the study and gave written informed consent at the baseline and follow-up evaluations.

## 3. Results

In this sample, 50.1% of the children were male, the median BMI *z*-score was 0.7 (IQR: 0–1.6), 95.6% had a gestational age older than 36 weeks, and 85.4% had a birth weight of 2500–3800 g. The median daily intake of cariogenic food and drinks was 2.7 (IQR: 1.9–4.0) and 1.8 (IQR: 1.9–3.1), respectively. The levels of 25(OH) D were inferior to 30 ng/mL in 65.1% of the sample. The mothers’ median age was 29.0 years (IQR: 25–33), their median BMI was 23.1 kg/m^2^ (IQR: 21.2–26.4), 48.0% had completed less than ten schooling years, and 38.8% had a monthly income greater than 1500 € (Table 1).

Regarding caries outcomes, no significant differences were found between the mixed dentition and the permanent teeth groups for the children’s sex, birth weight, gestation age and *z*-BMI. Children with dental caries status or advanced dental caries in permanent teeth had younger mothers, a lower monthly family income, and a higher prevalence of vitamin D levels below 30 ng/mL than children with no caries in these teeth (Table 1). Children with advanced dental caries in the mixed dentition had mothers with a higher BMI than children with no advanced dental caries, but no statistical differences were found in maternal BMI for dental caries status. Significantly more mothers of children with dental caries status or advanced dental caries had completed less than ten schooling years compared to mothers of children without caries. Similarly, on average, the daily frequency of cariogenic food and drinks intake was higher in children with dental caries status or advanced dental caries in mixed dentition and permanent teeth than in children with no caries (Table 1).

Regarding the potential effect of children’s weekly minutes spent reading and watching TV on their vitamin D levels, no association was found. On the other hand, we found a significant positive correlation (*p* = 0.031) between children’s outdoor activities and vitamin D levels, but its correlation coefficient was 0.118, which indicates that the correlation is negligible and, thus, has no clinical relevance (Table S2). Therefore, the time spent in these activities was not considered when assessing variables related to dental caries or interacting with vitamin D levels and dental caries.

The prevalence of dental caries status was 64.5% in the mixed dentition and 23.9% in permanent teeth. The prevalence of advanced dental caries was 62.4% in the mixed dentition and 20.0% in permanent teeth (Table 2). Nearly one-quarter of the children showed dental caries status in permanent teeth when incipient and cavitated lesions were considered, and one-fifth when only cavitated lesions were included. The prevalence of dental caries status and advanced dental caries was higher in both the permanent teeth and mixed dentition of children with 25(OH) D levels <30 ng/mL (Table 2).

The association of dental caries status and advanced dental caries with the children’s vitamin D levels, in our sample, was not affected by the season of blood collection (Table 3). In crude models, children with 25(OH) D levels <30 ng/mL at 7 years of age, when compared with those with 25(OH) D levels ≥30 ng/mL, had a significantly higher frequency of dental caries (OR = 2.00; 95%CI: 1.13–3.56; *p* = 0.018) and advanced dental caries (OR = 1.93; 95%CI: 1.04–3.56; *p* = 0.036) in permanent teeth (Table 3).

Regarding exposure variables in mixed dentition and permanent teeth, dental caries and advanced dental caries were associated with mothers who completed <10 and 10–12 schooling years, children with gastrointestinal disorders, having had a dental appointment at ≤7 years old, toothbrushing <1 time per day, higher daily intake of cariogenic food, higher daily intake of cariogenic drinks, and children’s vitamin D levels <30 ng/mL (Table S3).

The adjustment for maternal education did not attenuate the association between dental caries status in permanent teeth (D_1–6_MFT > 0) and the following independent variables: higher daily intake of cariogenic drinks and having had a dental appointment at ≤7 years old. The association between vitamin D levels <30 ng/mL and dental caries status in permanent teeth was not significant in the final multivariate logistic regression model (OR = 1.64 (95%CI: 0.87–3.03); *p* = 0.127). Nonetheless, remarkably, this analysis indicated a significant adjusted association between vitamin D levels <30 ng/mL and advanced dental caries in permanent teeth (OR = 2.27 (95%CI: 1.05–5.00); *p* = 0.037). A higher daily intake of cariogenic food, having had a dental appointment at ≤7 years old and children with gastrointestinal disorders were also factors associated with advanced dental caries in permanent teeth after adjusting for maternal education (Table 4). The multivariate logistic regression analysis indicated a significant unadjusted and adjusted association between vitamin D levels <30 ng/mL and the presence of advanced dental caries in permanent teeth.

Regarding the mixed dentition, the same independent variables were associated with dental caries status (d_1–6_mft and D_1–6_MFT > 0) and advanced dental caries (d_3–6_mft and D_3–6_MFT > 0) after adjustment for maternal education. The daily intake of more than one cariogenic food and having had a dental appointment at ≤7 years old were associated with dental caries status and advanced dental caries in the mixed dentition. No statistically significant association was observed between vitamin D levels or the interaction term (vitamin D < 30 ng/mL × winter season) and dental caries status and advanced dental caries in the mixed dentition (Table 4).

## 4. Discussion

In this study, we analysed the relationship between serum 25(OH) D levels and dental caries status and advanced dental caries in the mixed dentition and permanent teeth of a convenience sample of Portuguese children. Our primary finding was that 25(OH) D levels <30 ng/mL were associated with dental caries in the permanent teeth of 7-year-old children. This association between vitamin D threshold and advanced dental caries was not attenuated after adjusting for maternal education. Moreover, children with vitamin D levels ≥30 ng/mL showed a significantly lower proportion of dental caries and advanced caries in the mixed dentition. Nevertheless, serum 25(OH) D concentrations were not significantly correlated with caries in the mixed dentition both in the adjusted and the unadjusted models.

This study followed a rigorous methodology, aiming to bridge limitations detected in similar studies published in this area [30]. It also included variables not previously studied that could affect the relationship between vitamin D and dental caries, such as children’s activities and the season of blood sample collection.

Limited data on vitamin D concentrations among the European paediatric population are available from several countries [9]. A recent systematic review verified that, despite the abundance of solar UVB radiation in the Southern Europe and Eastern Mediterranean regions, more than one-third of the studies reported mean 25(OH) D levels <20 ng/mL. That systematic review highlighted an evident vitamin D deficiency across all population subgroups, which was highest among neonates/infants and adolescents, who go through critical periods of bone and overall growth and development [41]. The mean level of 25(OH) D (standard deviation) in our whole sample was 27.9 (8.2) ng/mL. Considering the seasons, the children’s mean 25(OH) D concentration was 30.2 (8.8) ng/mL in the summer months and 24.3 (5.5) ng/mL in the winter months. When comparing the 25(OH) D levels of our Portuguese sample with other Southern Europe countries, children from Spain (Pamplona and Asturias), Italy (Tuscany, Florence and Verona) and Turkey (Istanbul) had 25(OH) D levels significantly lower than our children sample. Even if stratified by season, Istanbul’s mean levels of vitamin D are significantly lower than those observed in our sample in both the summer and winter months [41]. Interestingly, in our sample, the season of blood collection (summer vs. winter) did not affect the association between dental caries and vitamin D levels. This finding may result from children having insufficient sun exposure, even in summer months, due to a more sedentary life, and using excessive amounts of high-factor sunscreen when in the sun, following skin cancer prevention campaigns [39].

Despite advances in prevention and management, 60% to 90% of schoolchildren experience dental caries, potentially resulting in pain, infection, and hospitalisation [1]. Dental caries is one of the most common diseases observed in paediatric patients worldwide [42], and thus, understanding the underlying mechanisms that relate early life events to a later occurrence of carious lesions will be key to develop current and more holistic long-term dental-caries preventive strategies in the future.

A growing body of evidence has reported vitamin D may help in preventing dental caries through its role in enamel and dentin formation [43] and induction of defensins and cathelicidins, which have antimicrobial properties [12]. Furthermore, interventions that provide adequate levels of vitamin D are theorised to reduce the prevalence of dental caries in children, affecting other health outcomes [24].

Herzog et al. reported no significant association between different vitamin D levels and dental caries in the mixed dentition of noninstitutionalised children aged 5 to 12 years in the United States [24]. These results are in line with ours regarding the experience of advanced dental caries in the mixed dentition since these authors did not consider incipient caries lesions. Dudding et al. found no evidence of an inverse causal effect of vitamin D on dental caries but found an association between low vitamin D and early caries onset [44]. On the other hand, Schroth et al. [20], who also examined the association between vitamin D levels and dental caries experience in the mixed dentition stage in a representative sample of Canadian children aged 6 to 11 years, suggested that optimal vitamin D concentrations (≥30 ng/mL) were associated with 39% lower odds of dental caries and dmft/DMFT in young school-aged children [20]. Our results for mixed dentition did not corroborate this finding. Furthermore, a recent randomised controlled trial reported no relationship between lower vitamin D levels and a higher risk of dental caries in permanent and primary teeth. Nevertheless, a high dose of vitamin D supplementation during pregnancy was associated with approximately 50% reduced odds of enamel defects in the offspring at 6 years of age [22].

In our sample, optimal children’s 25(OH) D levels (≥30 ng/mL) were associated with 56% lower odds of advanced dental caries in permanent teeth. These results are in agreement with the conclusions of Grant’s review, which suggested that optimal 25(OH) D levels were protective against caries [12]. Kühnisch et al. found that higher serum 25(OH) D values were associated with a reduced incidence of caries in permanent teeth [29]. This finding is in line with our results concerning advanced dental caries in permanent teeth since their results are related to cavitated dental lesions. Kim et al.’s results also agree with ours, as they found that children with low vitamin D levels had a higher proportion of caries in permanent teeth, mainly in permanent first molars [45], which represent the majority of permanent teeth assessed in our study.

Other covariates that were associated with caries in this sample included gastrointestinal disorders, cariogenic foods and drinks, and children having had a dental visit at ≤7 years old. Frequent consumption of excess amounts of sugar-sweetened beverages is a risk factor for obesity, type-2 diabetes, cardiovascular disease and dental caries [46]. Llena and Calabuig verified that a cariogenic diet, especially soft drinks, was associated with a high overall DMFT score and a high DMFT score only in first molars [47]. Our results agree with these findings.

Infrequent dental visits have been associated with an increased risk of untreated dental caries [48]. In the current study, children who had had a dental appointment at ≤7 years old had higher odds of having dental caries in both permanent teeth and mixed dentition than children who had never been to the dentist. This surprising finding might result from parents having sought dental care before because their child already had dental caries with treatment needs. The importance of preventive dental care in young children had not yet been instilled in caregivers. Our findings are in agreement with previous studies [20,49].

Our results should be interpreted considering the eruption period of all teeth that children present in their oral cavity at 7 years old. At this age, children have all primary teeth, which erupted between 6 months and 3 years of age, and permanent teeth that erupted between 6 and 7 years old. At 7 years old, the primary teeth have been more exposed than the recently erupted permanent teeth to other risk factors for caries, such as intake of sugar-sweetened beverages and foods and oral hygiene habits, and, thus, it is not surprising that the association between vitamin D and overall caries disappears over time. Accordingly, it is hypothesised that other risk factors for caries may overlap the possible preventive effect of vitamin D in the dental caries process. Therefore, low vitamin D levels may be related to caries only in permanent teeth that have recently erupted.

One of the major strengths of this study was the use of circulating serum 25(OH) D levels, measured by a reliable assay, as this is the best indicator of total vitamin D from both endogenous and exogenous sources [17]. Furthermore, the inclusion of intervening variables not previously considered clearly contributed to a broader and integrated view on this issue. Another strength of this study was the adjustment of this association for maternal education. Maternal education has a direct effect on children’s dental caries experience [50,51] and naturally influences socioeconomic status, which is a well-recognised social determinant of children’s oral health [52]. Not only do socioeconomic factors influence oral health, but they also may place the children at risk of poor nutrition, thereby possibly impacting their 25(OH) D levels [7].

Another distinctive aspect that contributes to the quality of our study is the method used to record dental caries, based on the ICDAS II. Using this system, we categorised lesions as cavitated and non-cavitated and conducted a complex analysis based on the severity of the lesions that is not generally conducted in other investigations. The separate analysis of the mixed dentition and the permanent teeth also allowed obtaining more reliable results, considering that primary and permanent teeth erupt at different ages and, thus, the time of exposure to other caries risk factors could confound the results’ analysis. Moreover, trained, calibrated dentists performed the dental examinations, which improved consistency in procedures and registration of the caries diagnosis, and they were blinded to the children’s 25(OH) D levels. In our sample, the proportion of dental caries in the mixed dentition was within the expected range based on the third national survey performed in Portugal. However, dental caries prevalence in permanent teeth was much higher in our sample, from Porto, than in children from Northern Portugal in the national epidemiological survey [53,54].

A limitation of this study is the cross-sectional nature of data, which does not allow us to determine causality. This type of study design does not provide any prior knowledge of children’s vitamin D status at the time their teeth were developing [29]. However, knowing that vitamin D levels may not change dramatically during childhood, those children with adequate and optimal levels of 25(OH) D at 7 years old likely had beneficial concentrations in the past, during the previous period of permanent tooth development, thereby ensuring proper dental development of enamel and dentin that would be more resistant to caries [20]. Another limitation of this study is not including a randomly selected sample but rather a convenience one and, so, the generalizability of our findings may be limited. Dental examinations did not include radiographs and, therefore, the results may underestimate the true prevalence of untreated caries and restorations. We also recognise that including additional information related to children’s prematurity, type of medication used, and diseases could have allowed a more accurate analysis of these variables’ influence on the levels of vitamin D and, thus, contribute to obtaining more accurate results.

The findings of this study suggest that 25(OH) D levels <30 ng/mL are associated with dental caries in permanent teeth. On the other hand, children’s optimal 25(OH) D concentrations are associated with 56% lower odds of advanced dental caries in permanent teeth. Our findings were supported by a rigorous methodology, providing consistent and reliable results. Therefore, considering that vitamin D may influence oral health, its importance in preventing children’s dental caries should be reinforced. Early childhood oral health policies must always focus on preventive measures regarding behavioural caries risk factors, but improving children’s nutrition and interventions that provide adequate levels of vitamin D should also be considered as a priority.

## 5. Conclusions

Based on the results of this study, children’s 25(OH) D levels <30 ng/mL are associated with advanced dental caries in permanent teeth in 7-year-old children. In the mixed dentition, other social and behavioural factors appear to be associated with both dental outcomes in our Portuguese sample. Optimal levels of vitamin D in childhood may be considered an additional preventive measure for dental caries in the permanent dentition.

## Figures and Tables

**Table 1:** Mothers’ and children’s characteristics for the whole sample and distributed per children with dental caries status (caries vs. no caries) in the mixed dentition and permanent teeth, and children with advanced dental caries (advanced caries vs. no advanced caries) in the mixed dentition and permanent teeth.

				Dental Caries Status						Advanced Dental Caries					
	All Children (*n* = 335)			Mixed Dentition			Permanent Teeth			Mixed Dentition			Permanent Teeth		
Characteristics	*n*	%	Median (IQR)	Yes ^¥^	No ^¥¥^	*p* *	Yes ^‡^	No ^‡‡^	*p* *	Yes ^Ø^	No ^ØØ^	*p* *	Yes ^§^	No ^§§^	*p* *
**Mothers’ characteristics**															
**Mothers’ age (*n* = 335) (years)**															
median (IQR)			29.0 (25–33)	28.5 (24–33)	29.0 (26–33)	0.066	27.0 (23–31.8)	29.0 (26–33)	**0.013**	28.0 (24–33)	29.0 (26–33)	0.082	27.0 (22–31)	29.0 (25.25–33)	**0.008**
**Mothers’ BMI (*n* = 300) (kg/m^2^)**															
mean (SD)			23.1 (21.2–26.4)	23.4 (21.3–26.9)	22.7 (20.8–25.7)	0.056	23.2 (21.2–26.4)	23.1 (21.1–26.4)	0.957	23.5 (21.3–26.9)	22.6 (20.8–25.7)	**0.026**	23.2 (21.3–26.3)	23.1 (21.1–26.5)	0.805
**Monthly income (*n* = 325)**															
Low: ≤1000 €	108	33.2		76 (36.4)	32 (27.6)	0.055	38 (48.7)	70 (28.3)	**0.003**	72 (35.6)	36 (29.3)	0.052	30 (46.2)	78 (30.0)	**0.042**
Intermediate: 1001–1500 €	91	28.0		62 (29.7)	29 (25.0)		19 (24.4)	72 (29.1)		62 (30.7)	29 (23.6)		16 (24.6)	75 (28.8)	
High: >1500 €	126	38.8		71 (34.0)	55 (47.4)		21 (26.9)	105 (42.5)		68 (33.7)	58 (47.2)		19 (29.2)	107 (41.2)	
**Maternal education (*n* = 333)**															
≤9 years	160	48.0		114 (53.3)	46 (38.7)	**0.002**	51 (43.1)	109 (43.1)	**0.001**	111 (53.6)	49 (38.9)	**0.003**	43 (64.2)	117 (44.0)	**0.004**
10–12 years	101	30.3		66 (30.8)	35 (29.4)		23 (28.8)	78 (30.8)		63 (30.4)	38 (30.2)		18 (26.9)	83 (31.2)	
>12 years	72	21.6		34 (15.9)	38 (31.9)		6 (7.5)	66 (26.1)		33 (15.9)	39 (31.0)		6 (9.0)	66 (24.8)	
**Children’s characteristics**															
**Sex (*n* = 335)**															
Female	167	49.9		105 (48.6)	62 (52.1)	0.541	36 (45.0)	131 (51.4)	0.320	100 (47.8)	67 (53.2)	0.345	31 (46.3)	136 (50.7)	0.512
Male	168	50.1		111 (51.4)	57 (47.9)		44 (55.0)	124 (48.6)		109 (52.2)	59 (46.8)		36 (53.7)	132 (49.3)	
**Birth weight (*n* = 335)**															
<2500 g	18	5.4		12 (5.6)	6 (5.0)	0.497	6 (7.5)	12 (4.7)	0.539	12 (5.7)	6 (4.8)	0.628	5 (7.5)	13 (4.9)	0.698
2500–3800 g	286	85.4		187 (86.6)	99 (83.2)		68 (85.0)	218 (85.5)		180 (86.1)	106 (84.1)		56 (83.6)	230 (85.8)	
>3800 g	31	9.3		17 (7.9)	14 (11.8)		6 (7.5)	25 (9.8)		17 (8.1)	14 (11.1)		6 (9.0%)	25 (9.3)	
**Gestational age (*n* = 271)**															
<37 weeks	12	4.4		8 (4.7)	4 (4.0)	**1.000**	4 (6.1)	8 (3.9)	0.494	8 (4.8)	4 (3.8)	0.771	3 (5.7)	9 (4.1)	0.708
≥37 weeks	259	95.6		164 (95.3)	95 (96.0)		62 (93.9)	197 (96.1)		158 (95.2)	101 (96.2)		50 (94.3)	209 (95.9)	
**z-BMI (*n* = 334)**															
median (IQR)			0.7 (0–1.6)	0.7 (−0.1–1.8)	0.8 (0.1–1.5)	0.764	0.7 (0.0-2.0)	0.7 (−0.1–1.6)	0.679	0.7 (−0.1–1.8)	0.7 (0.1–1.5)	0.681	0.8 (0.0–2.2)	0.7 (−0.1–1.6)	0.264
**Daily intake of cariogenic food (*n* = 333)**															
median (IQR)			2.7 (1.9–4.0)	3.1 (2.1–4.3)	2.3 (1.7–3.3)	**<0.001**	3.0 (2.1–4.3)	2.6 (1.9–3.9)	**0.050**	3.1 (12.1–4.3)	2.3 (1.7–3.4)	**<** **0.001**	3.1 (2.2–4.3)	2.5 (1.9–3.9)	**0.016**
**Daily intake of cariogenic drink (*n* = 333)**															
median (IQR)			1.8 (1.0–3.1)	2.1 (1.3–3.2)	1.3 (0.8–3.0)	**0.002**	2.4 (1.4–3.6)	1.7 (1.0–3.0)	**0.004**	2.1 (1.3–3.2)	1.4 (0.8–3.0)	**0.005**	2.3 (1.3–3.5)	1.7 (1.0–3.1)	**0.020**
**Vitamin D (ES ref.) (*n* = 335)**															
<30 ng/mL	218	65.1		145 (67.1)	73 (61.3)	0.288	61 (76.3)	157 (61.6)	**0.016**	142 (67.9)	76 (60.3)	0.156	51 (76.1)	167 (62.3)	**0.034**
≥30 ng/mL	117	34.9		71 (32.9)	46 (38.7)		19 (23.8)	98 (38.4)		67 (32.1)	50 (39.7)		16 (23.9)	101 (37.7)	

Bold entries denote statistical significance (*p* < 0.05). * *p*-value: Two-sample Student *t*-test, Mann–Whitney U-test, chi-square test or Fisher’s exact test, as appropriate; *p* < 0.001 (Bonferroni correction); Yes ^¥^, d_1–6_mft/D_1–6_MFT > 0: dental caries in mixed dentition; No ^¥¥^, d_1–6_mft/D_1–6_MFT = 0: no dental caries in mixed dentition; Yes ^‡^, D_1–6_MFT > 0: dental caries in permanent teeth; No ^‡‡^, D_1–6_MFT = 0: no dental caries in permanent teeth; Yes ^Ø^, d_3–6_mft/D_3–6_MFT > 0: advanced dental caries in mixed dentition; No ^ØØ^, d_3–6_mft/D_3–6_MFT = 0: no advanced dental caries in mixed dentition; Yes ^§^, D_3–6_MFT > 0: advanced dental caries in permanent teeth; No ^§§^, D_3–6_MFT = 0: no advanced dental caries in permanent teeth; ES ref., Endocrine Society reference; IQR, Interquartile Range.

**Table 2:** Prevalence of dental caries status and advanced dental caries in the mixed dentition and the permanent teeth for the whole sample and according to vitamin D reference values.

	All Children (*n* = 335)		25(OH) D <30 ng/mL		25(OH) D ≥30 ng/mL	
	*n* (%)	95%CI	*n* (%)	95%CI	*n* (%)	95%CI
**Dental Caries Status**						
Mixed Dentition	216 (64.5)	59.4–69.6	145 (66.5)	60.3–72.8	71 (60.7)	51.8–69.5
Permanent Teeth	80 (23.9)	19.3–28.5	61 (28.0)	22.0–33.9	19 (16.2)	9.6–22.9
**Advanced Dental Caries**						
Mixed Dentition	209 (62.4)	57.2–67.6	142 (65.1)	58.8–71.5	67 (57.3)	48.3–66.2
Permanent Teeth	67 (20.0)	15.7–24.3	51 (23.4)	17.8–29.0	16 (13.7)	7.5–19.9

**Table 3:** Crude association of dental caries status and advanced dental caries with the children’s vitamin D levels and the interaction between vitamin D levels and season.

	Dental Caries Status		Advanced Dental Caries	
	OR	95% CI	OR	95% CI
**Permanent Teeth**				
25(OH) D levels (<30 ng/mL)	**2.00**	**1.13** **–** **3.56**	**1.93**	**1.04** **–** **3.56**
**Mixed Dentition**				
Season (winter) by 25(OH) D levels (<30 ng/mL)	1.47	0.90–2.40	1.49	0.92–2.40

Bold entries denote statistical significance (*p* < 0.05). Variable(s) entered on step 1: Season*Vit.D < 30 ng/mL; and vit. D < 30 ng/mL OR, odds ratio; CI, confidence interval; ng/mL, nanograms per millilitre. Reference categories: 25(OH) D levels <30 ng/mL for dental caries status and advanced dental caries in permanent teeth; interaction between the winter season and 25(OH) D <30 ng/mL for dental caries status and advanced dental caries in mixed dentition.

**Table 4:** Association (multivariate logistic regression model) between dental caries status and advanced dental caries in permanent teeth and mixed dentition and the mothers’ and children’s characteristics.

	Permanent Teeth		Mixed Dentition	
	Dental Caries Status *	Advanced Dental Caries *	Dental Caries Status *	Advanced Dental Caries *
	OR (95% CI)	OR (95% CI)	OR (95% CI)	OR (95% CI)
**Gastrointestinal disorders** (Yes)	3.23 (0.78–13.44)	**5.01 (1.13–22.15)**	-	-
**Cariogenic drinks** (+1 item per day)	**1.20 (1.02–1.41)**	-	-	-
**Cariogenic foods** (+1 item per day)	-	**1.20 (1.03–1.40)**	**1.41 (1.18–1.68)**	**1.39 (1.18–1.65)**
**Dental appointment** (Yes)	**2.57 (1.16–5.68)**	**3.21 (1.26–8.22)**	**2.94 (1.61–5.39)**	**2.68 (1.47–4.89)**
**Vitamin D** (<30 ng/mL)	1.64 (0.87–3.03)	**2.27 (1.05–5.00)**	-	-
**Season by Vitamin D levels** (winter **x** < 30 ng/mL)	-	2.93 (0.80–10.83)	0.52 (0.20–1.39)	-

Bold entries denote statistical significance (*p* < 0.05). * The multivariate logistic regression models adjusted for maternal education. OR, odds ratio; CI, confidence interval; ng/mL, nanograms per millilitre.

## Data Availability

Data used in this study were from the Generation XXI birth cohort and it is under the responsibility of Professor Henrique Barros, head of the Department of Public Health and Forensic Sciences, and Medical Education of the University of Porto Medical School, and president of the Institute of Public Health of the University of Porto. For the present study, individual-level information was used, that cannot be disseminated due to confidentiality issues. A formal request to the person responsible (Professor Henrique Barros: hbarros@med.up.pt) can be made by anyone interested in developing scientific research based on data collected within the Generation XXI study. Further information can be found at the Institute of Public Health website: http://ispup.up.pt/research/research-structures/.

## Supplementary Materials

The following are available online at https://www.mdpi.com/2072-6643/13/1/166/s1, Table S1: Comparison between the characteristics of the eligible participants and the remaining cohort evaluated at baseline^*^ (Number of participants and percentages; median and interquartile range). Table S2: Dose-response association between 25(OH) D levels and children’s activities at 7 years of age (children’s weekly minutes spent reading, watching TV, and doing outdoor activities). Table S3: Bivariate analysis between dental caries and advanced dental caries in mixed dentition and permanent teeth with independent (exposure) variables.
