# Supplementary material for: Serum Levels of Vitamin D and Dental Caries in 7-Year-Old Children in Porto Metropolitan Area
Source: Nutrients. 2021 Jan 7;13(1):166. doi: 10.3390/nu13010166 (PMC7825719; doi:10.3390/nu13010166)
Supplement: Supplementary file 1 [file nutrients-13-00166-s001.pdf]

Table S1. Comparison between the characteristics of the eligible participants and the remaining cohort evaluated at baseline* (Number of participants and percentages; median and interquartile range).

	**Sample[¥] (n = 335)**		**Remaining Cohort[§] (n = 8312)**		
	**n**	**%**	**n**	**%**	*p*
**Gestational age n (%)**					
<37 weeks	12	4.4	616	9.4	
≥37 weeks	259	95.6	5971	90.6	**0.006**
**Weight at birth** (g)					
Median	3240		3190		
(IQR)	(2910-3530)		2880-3490		0.074
**Mothers' age** (years)					
Median	29		29		
(IQR)	(25-33)		(25-33)		0.536
**Maternal education n (%)**					
≤9 years	160	48.0	4068	49.3	
10-12 years	101	30.3	2199	26.6	0.278
>12 years	72	21.6	1991	24.1	
**Monthly household income n (%)**					
Low: ≤1000€	108	33.2	2021	31.8	
Intermediate: 1001-1500€	91	28.0	1750	27.6	0.906
High: >1500€	126	38.8	2575	40.6	

*For each variable, the total may not add to 335/8312 due to missing data.

¥Children who attended the dental examination at 7 years of age, had permanent teeth erupted and had a blood sample collected.

§Cohort evaluated at baseline.

IQR, Interquartile Range

Scheme S2. Table. Dose-response association between 25(OH) D levels and children's activities at 7 years of age (children's weekly minutes spent reading, watching TV, and doing outdoor activities).

	**Vitamin D (ng/ml) vs Reading (min/week)**	**Vitamin D (ng/ml) vs Watching TV (min/week)**	**Vitamin D (ng/ml) vs Reading and watching TV (min/week)**	**Vitamin D (ng/ml) vs Doing outdoor activities (min/week)**
**n**	334	334	334	333
p	0.625	0.300	0.194	**0.031**
$\mathbf{r_s}$	-0.027	-0.057	-0.071	0.118

Bold entries denote statistical significance ($p<0.05$).

$\mathbf{r_s}$, Spearman's correlation coefficient.

Table S3. Bivariate analysis between dental caries and advanced dental caries in mixed dentition and permanent teeth with independent (exposure) variables.

	**Dental Caries Status**						**Advanced Dental Caries**					
	**Mixed Dentition (d_{1-6}mft and D_{1-6}MFT)**			**Permanent Teeth (D_{1-6}MFT)**			**Mixed Dentition (d_{3-6}mft and D_{3-6}MFT)**			**Permanent Teeth (D_{3-6}MFT)**		
	**No n (%)**	**Yes n (%)**	***p***	**No n (%)**	**Yes n (%)**	***p***	**No n (%)**	**Yes n (%)**	***p***	**No n (%)**	**Yes n (%)**	***p***
**Gestational age**												
<37 weeks	4 (4.0)	8 (4.7)	1.000	8 (3.9)	4 (6.1)	0.494	4 (3.8)	8 (4.8)	0.771	9 (4.1)	3 (5.7)	0.708
≥37 weeks	95 (96)	164 (95.3)		197 (96.1)	62 (93.9)		101 (96.2)	158 (95.2)		209 (95.9)	50 (94.3)	
**Weight at birth**												
n	119	216		255	80		126	209		268	67	
Median	3215	3250	0.505	3255	3190	0.808	3212.5	3250	0.519	3250	3230	0.707
(IQR)	(2790-3535)	(2962.8-3512.5)		(2850-3530)	(3012.5-3478.8)		(2807.5-3531.3)	(2957.5-3525)		(2882.5-3530)	(3000-3485)	
**Maternal education**												
≤ 9 years	46 (38.7)	114 (53.3)		109 (43.1)	51 (63.8)		49 (38.9)	111 (53.6)		117 (44.0)	43 (64.2)	
10-12 years	35 (29.4)	66 (30.8)	**0.002**	78 (30.8)	23 (28.8)	**0.001**	38 (30.2)	63 (30.4)	**0.003**	83 (31.2)	18 (26.9)	**0.004**
>12 years	38 (31.9)	34 (15.9)		66 (26.1)	6 (7.5)		39 (31.0)	33 (15.9)		66 (24.8)	6 (9.0)	
**Vitamin and drugs intake**												
No	99 (83.9)	181 (84.6)	0.870	213 (83.9)	67 (85.9)	0.665	103 (82.4)	177 (85.5)	0.450	223 (83.8)	57 (86.4)	0.613
Yes	19 (16.1)	33 (14.5)		41 (16.1)	11 (14.1)		22 (17.6)	30 (14.5)		43 (16.2)	9 (13.6)	
**Bone fracture**												
No	114 (95.8)	199 (92.6)	0.243	237 (92.9)	76 (96.2)	0.297	120 (95.2)	193 (92.8)	0.371	249 (92.9)	64 (97.0)	0.224
Yes	5 (4.2)	16 (7.4)		18 (7.1)	3 (3.8)		6 (4.8)	15 (7.2)		19 (7.1)	2 (3.0)	
**Gastrointestinal disorders**												
No	114 (96.6)	208 (97.2)	0.748	249 (98)	73 (93.6)	0.059	120 (96.0)	202 (97.6)	0.511	261 (98.1)	61 (92.4)	0.030
Yes	4 (3.4)	6 (2.8)		5 (2.0)	5 (6.4)		5 (4.0)	5 (2.4)		5 (1.9)	5 (7.6)	
**Kidney disease**												
No	113 (95)	210 (98.1)	0.176	244 (96.1)	79 (100)	0.125	119 (94.4)	204 (98.6)	**0.046**	256 (96.2)	67 (100)	0.222
Yes	6 (5.0)	4 (1.9)		10 (3.9)	0 (0.0)		7 (5.6)	3 (1.4)		10 (3.8)	0 (0.0)	
**Muscle disorders**												
No	111 (94.9)	208 (97.2)	0.357	243 (96.0)	76 (97.4)	0.739	118 (95.2)	201 (97.1)	0.376	254 (95.8)	65 (98.5)	0.472
Yes	6 (5.1)	6 (2.8)		10 (4.0)	2 (2.6)		6 (4.8)	6 (2.9)		11 (4.2)	1 (1.5)	
**Has the child ever been to the dentist?**												
No	35 (30.7)	36 (17.6)	**0.007**	58 (24.2)	13 (16.5)	0.153	36 (30.0)	35 (17.6)	**0.010**	63 (24.9)	8 (12.1)	**0.026**
Yes	79 (69.3)	169 (82.4)		182 (75.8)	66 (83.5)		84 (70.0)	164 (82.4)		190 (75.1)	58 (87.9)	
**Toothbrushing frequency**												
< 1 time a day	35 (29.4)	83 (40.1)		89 (35.3)	29 (39.2)		37 (29.4)	81 (40.5)		93 (35.2)	25 (40.3)	
1 time a day	35 (29.4)	56 (27.1)	0.136	67 (26.6)	24 (32.4)	0.295	36 (28.6)	55 (27.5)	0.088	69 (26.1)	22 (35.5)	0.088
≥ 2 times a day	49 (41.2)	68 (32.9)		96 (38.1)	21 (28.4)		53 (42.1)	64 (32.0)		102 (38.6)	15 (24.2)	
**Cariogenic food intake**												
n	119	214		255	78		126	207		267	66	
Median	2.3	3.1	**<0.001**	2.6	3.0	**0.050**	2.3	3.1	**<0.001**	2.5	3.1	**0.016**
(IQR)	(1.7-3.3)	(2.1-4.3)		(1.9-3.9)	(2.1-4.3)		(1.7-3.4)	(2.1-4.3)		(1.9-3.9)	(2.2-4.3)	
**Consumption of cariogenic drinks**												
n	119	214		255	78		126	207		267	66	
Median	1.3	2.1	**0.002**	1.7	2.4	**0.004**	1.4	2.1	**0.005**	1.7	2.3	**0.020**
(IQR)	(0.8-3.0)	(1.3-3.2)		(1.0-3.0)	(1.4-3.6)		(0.8-3.0)	(1.3-3.2)		(1.0-3.1)	(1.3-3.5)	

	Dental Caries Status						Advanced Dental Caries					
	Mixed Dentition (d_{1-6}mft and D_{1-6}MFT)			Permanent Teeth (D_{1-6}MFT)			Mixed Dentition (d_{3-6}mft and D_{3-6}MFT)			Permanent Teeth (D_{3-6}MFT)		
	**No n (%)**	**Yes n (%)**	***p***	**No n (%)**	**Yes n (%)**	***p***	**No n (%)**	**Yes n (%)**	***p***	**No n (%)**	**Yes n (%)**	***p***
**z-BMI**												
n	119	215		255	79		126	208		267	67	
Median	0.8	0.7	0.764	0.7	0.7	0.679	0.7	0.7	0.681	0.7	0.8	0.264
(IQR)	(0.1-1.5)	(-0.1-1.8)		(-0.1-1.6)	(0.0-2.0)		(0.1-1.5)	(-0.1-1.8)		(-0.1-1.6)	(0.0-2.2)	
**Vitamin D**												
n	119	216		255	80		126	209		268	67	
Median	27.0	26.0	0.425	27.0	25.0	0.086	27.0	26.0	0.233	27.0	25.0	0.088
(IQR)	(22.0-32.0)	(22.3-32.0)		(22.0-32.0)	(22.3-29.0)		(22.0-32.3)	(22-32.0)		(22.0-32.0)	(22.0-29.0)	

Bold entries denote statistical significance ($p<0.05$). Data are presented as proportions (%) for categorical measures and median values and interquartile range for continuous measures. Differences between groups were tested with Fisher's exact test or chi-square test, respectively.
